# Dental Chemistry

**Published:** 1872-12

**Authors:** L. G. Noel


### Dental Register.

VOL. XXVI.] DECEMBER, 1872. No. 12.]

### COMMUNICATIONS.

### Dental Chemistry.

BY DR. L. G. NOEL.

*Read before the Tennessee State Dental Society.*

The explored regions of chemistry are indeed wide, but the measure of the vast immensity yet unknown, what mind can grasp? Would that I could enter these rich fields and pluck a flower of truth suitable to deck the brow of our Mistress on this occasion, but I must be content to make such offering as I have. You Gentlemen, join in the general shout for something new, in soliciting this report; but I have no new theories, perhaps no new discoveries for you. Permit me then, in a rambling way, to present for your consideration a few thoughts that of late have occupied my mind, but which, for want of time, I must bring before you in a crude, unfinished state. In the first place, I wish to call attention to the subject of alkalies, as agents in the production of caries of the teeth. My thoughts were turned into this channel recently by a conversation had with my friend, Prof. Summers, who it will be remembered, was a strong advocate for the alkaline theory, at the last meeting of the American Dental Association, at Niagara. His theory as presented to me, was so plausible and clear, that I went into the investigation of the subject with great enthusiasm in its favor. His views are briefly as follows: Alkalies are more destructive in their action upon the teeth than acids. They dissolve out the gelatinous matrix, leaving the lime salts without support, and in a disintegrating condition. Dr. H. L. Sage, in a paper read before the American Dental Association at its recent meeting, advanced substantially the same views; and from the brief report of the proceedings, which appear in the *Dental Cosmos*, it would seem that they met with no very great opposition. So recently has my attention been turned to this matter, that I have not had time to investigate it as thoroughly as I could wish. The simple experiments which I have made, were begun with the expectation of substantiating the views above stated, so that I could report definitely at this meeting; but the results, I confess, have not been what I expected, and I present them for what they are worth. They were conducted in the following manner: three microscopic sections of teeth, including dentine and enamel, were treated with three different alkalies; thus, No. 1, with aqua ammonia, 1 fluid drachm to one-half ounce water; No. 2, with caustic soda, 3 grains, to one-half ounce water; No. 3, caustic potash, 3 grains, to one-half ounce water. These sections were macerated, one in each of the above solutions, for twenty-four hours, at the temperature of the surrounding atmosphere. At the expiration of this time they were removed and dried. No change could be detected by the unaided eye. The polished surfaces were found undisturbed, and the sections had lost none of their elasticity and toughness. Under the microscope no change could be detected in the arrangement of the tissues. Three more sections were prepared, and treated with stronger solutions of the same alkalies, forty-eight hours, at the temperature of the surrounding atmosphere. This time the ammonia was the aqua ammonia of the shops, in full strength; the potash and soda being used in saturated solutions. Upon removing from the fluids no change could be detected, either with the unaided eye or microscope. Other sections were now prepared, and treated in the same manner, but with higher temperatures, with various results. No very marked change was produced, however, until the temperature was carried up to the boiling point. Ammonia was not tried at this temperature; but caustic potash and soda produced in thirty minutes quite a marked effect. The polished surfaces were roughened, the sections were found more brittle, and much more readily scrached with an excavator. Under the microscope small pits were observed where the dentinal tubuli appeared to end abruptly in an open space, and other points, where they had lost their definite arrangement, by the dissolution of the animal matrix. The results obtained by these experiments, were not nearly so marked as we had expected to see, and we confess, that our enthusiasm for the alkaline theory was lost; still it may be allowed, that they play some part in the production of decay. By boiling gelatine in strong alkalies it is converted into leucine and glycocine, with evolution of ammonia.

Glycocine possesses the properties of a base; and is found in the bile, united with cholic acid and soda, as the glycocholate of soda. This fact, would justify the inference that glycocine is formed by the disintegration of the gelatine of the tissues; but the possulate, that it arises by the union of the gelatine of the tissues with the alkalies of the fluids, is materially weakened, when we remember that it is also produced by the action of acids, on gelatinous substances. If we could have obtained any appreciable result, at a temperature equa to that of the mouth, we would have regarded our first views as in a great measure confirmed. We are willing to ascribe to alkalies the place of auxiliaries, in the production of decay; but until there is more positive proof to the contrary, must regard acids as the great agents of destruction. In one of the most rapid varieties of decay, we find the gelatinous portion left in the bottom of the cavity, as a tough leathery mass, after the lime salts are all dissolved out. Here, certainly, alkalies play no part. Frequently, however, this portion is dissolved out, as rapidly as decay advances; but its solution may be effected by the water of the saliva alone. It is a well-known fact that gelatine is soluble in water. Another matter to which we wish to call attention, was brought to our notice, by an article which appeared in the DENTAL REGISTER, in which the writer attributes the disintegration of oxychloride of zinc fillings to the presence of alkalies in the saliva. A case is cited, in which fillings of Guillois' cement rapidly disintegrated in the presence of alkaline saliva; and the writer, Dr. H. L. Sage, seems to attribute their rapid decomposition to the neutralization of the excess of acid, which was present in the chloride.

Guilloi's cement, and all that class of preparations, are composed chiefly of oxychloride of zinc. They probably contain a small percentage of silica, carbonate of lime, and other earthy matters, calculated to increase their density; but these are in the main held together by oxychloride of zinc. The white oxide of zinc, which these preparations contain, is prepared by heating the carbonate to redness, or by burning metallic zinc in the air. It contains one eq. of oxygen, and is formulated thus, Zn. O. The chloride is prepared by the action of chlorine on zinc, or by treating zinc with hydrochloric acid. Its formula is Zn. Cl. (Fowne has it Zn. Cl.2, but Miller and Gregory regard it as a protochloride.)

Now let us examine the changes which take place when oxychloride of zinc is brought in contact with acids and alkalies. If a piece of it is placed in a solution of caustic potash, caustic soda, or aqua ammonia, it will soon become very much softened, and finally its integrity is completely destroyed. This takes place much sooner in ammonia than any other alkali. The change is simply one of displacement, and not dependent upon the neutralization of the acid, as Dr. Sage supposes. If the alkali be soda, the chlorine unites with the sodium, forming chloride of sodium; the oxygen of the soda combines with the atom of zinc thus freed from the chlorine; and while chloride of sodium is held in solution the oxide of zinc remains unchanged in the bottom of the vessel. If the alkali be ammonia, salammoniac is formed, and two eq. of zinc oxide remains. A like result is obtained with potash.

The reaction may be represented thus: $Zn.\ O.+Zn.\ Cl.+K.\ O.\ H.\ O.=K.\ Cl.+2Zn.\ O.+H.\ O.$ or $Zn.\ O.+Zn.\ Cl.+N.\ H._4\ O.=N.\ H_4\ Cl.+2Zn.\ O.$

Acids are also destructive to its integrity. Thus if acetic or lactic acids exist in the saliva where this substance is used for fillings, the acetate or lactate of zinc will be formed, and hydrochloric acid will be envolved. This change may be made more comprehensible by the following formula: $Zn. O. + Zn. Cl. + C_6 H._5 O_5 + H. O. = H. Cl. + Zn. O. C._6 H._5 O._5 + Zn. O.$ These acids, lactic and acetic, are so frequently present in the saliva that we need hardly be surprised that oxychloride of zinc always fails, as a permanent filling. Dr. Wright, of Edinburgh, whose papers on saliva, published in the London *Lancet*, have been held in great esteem, assures us that free soda and albuminate of soda always exist in healthy saliva. Hence we have at all times, whether acid or alkaline, the elements essential to the decomposition of oxychloride of zinc in the saliva. Gentlemen, will you use it for permanent, fillings in the face of these facts?

